# The effect of socio-economic background on parental knowledge regarding oral health and its association with proactive behaviours

**DOI:** 10.1007/s40368-026-01168-0

**Published:** 2026-01-21

**Authors:** K. Seremidi, K. Chatzidimitriou, A. Theristopoulos, S. Gizani, W. Papaioannou

**Affiliations:** 1 Department of Paediatric Dentistry, School of Dentistry, National and Kapodistrian University of Athens, Athens, Greece; 2 Department of Preventive & Community Dentistry, School of Dentistry, National and Kapodistrian University of Athens, Athens, Greece

**Keywords:** Socio-economic status, Parental knowledge, Association, Oral health, Dental behaviours

## Abstract

**Purpose:**

To evaluate the effect of socio-economic background on oral health knowledge and cultural beliefs of parents and associate knowledge to proactive behaviours.

**Methods:**

This is a cross-sectional study with a convenient sample consisting of parents seeking dental care for their children at two different dental centres. Data were collected via a structured, interview-based questionnaire, covering demographic characteristics and parental oral health knowledge and behaviours. Analysis was performed based on parental socioeconomic background determined by monthly family income (< 1400€ low, 1400–2500€ medium). Differences within and between groups were assessed using chi-square and Fisher’s exact tests and associations with demographic or behavioural characteristics using multivariate regression analysis (statistical significance *p* < 0.05).

**Results:**

Of the participants, 111 were from low and 105 from medium socio-economic backgrounds, with the latter demonstrating significantly better oral health knowledge and more proactive behaviours, such as regular preventive dental visits and lower consumption of sugary snacks (*p* < 0.05). Parents from low socio-economic backgrounds showed poor knowledge regarding aetiopathogenesis of common dental diseases, with significant differences for the effect of microbes in dental plaque accumulation formation (*p* = *0.03*) and for brushing (*p* = *0.02*) and sugary snack consumption (*p* = *0.04*) in caries development. Multivariate regression analysis showed that area of residency and mother’s occupation were significantly associated with decreased knowledge, that was associated with infrequent brushing and frequent sugary snack consumption.

**Conclusion:**

Significant associations highlighted the need for targeted educational interventions and public health policies to reduce oral health disparities and improve awareness.

## Introduction

Oral health is an essential component of patients’ general health and overall well-being. Poor oral health in terms of irregular oral hygiene habits, imbalanced diet with frequent snacking, and infrequent dental visits is directly related to common dental diseases, including dental caries and periodontal disease. Advances in knowledge of the aetiopathogenesis of dental diseases have highlighted the importance of behavioural factors in their development and prevalence. Their effect is even greater in children, as their oral health is highly dependent on their parents’ knowledge, behaviours, cultural beliefs, and awareness regarding oral health (Glick et al. 2016).

Parents serve as primary role models, and their awareness of the importance of oral health can substantially impact their children’s oral health outcomes. Their knowledge is directly affected by factors such as level of education and socioeconomic status (SES), as determined by income, occupation, and social status (Braveman et al. 2005). These factors are key determinants in shaping parental behaviours towards oral and dental health. A social gradient in health is determined by socioeconomic disparities, with those who are economically and socially disadvantaged having worse oral and general health (Marmot and Wilkinson 2001), potentially due to the fact that these groups are more likely to be exposed to risk factors for the development of dental diseases (Singh et al. 2019). Socio-environmental factors might make it difficult for parents to implement their knowledge, particularly when unhealthy dietary options are widely available, easily accessible, convenient, more economical, and tastier. It has been shown that families from lower SES are more likely to create an environment that favours an unhealthy lifestyle (Hawkes et al. 2013).

It is, therefore, evident that improving parental awareness and behaviours is a crucial initial step in controlling children’s oral health. Targeted preventive programmes can help parents and children create a positive attitude towards their oral health, that will be maintained throughout their lives (Adair et al. 2004; Mattila et al. 2005). However, for preventive programmes to be more effective, it is essential to identify individuals who are at higher risk of developing dental disease. The World Health Organization has made suggestions to enhance preschoolers’ and school-aged children’s knowledge, behaviours, and actions regarding oral health to prevent and control oral diseases (Rad et al. 2015).

Like many other developed countries, Greece faces oral health disparities that disproportionately affect lower-income and immigrant populations. These vulnerable groups often encounter significant barriers to access dental care due to financial constraints, lack of education, and language barriers (Diamanti et al. 2021). Previous research in Greece suggests that children from such backgrounds are more likely to experience higher levels of dental caries and other oral health issues, further exacerbating health inequalities (Theristopoulos et al. 2024). Despite the well-documented influence of SES on oral health, limited research has been conducted to compare oral health knowledge and behaviours of parents from different socioeconomic backgrounds. Understanding these differences is essential for developing targeted public health strategies and educational programmes aimed at promoting better oral health practices amongst disadvantaged communities.

Therefore, the aim of the present study was to evaluate the effect of socio-economic background on oral health knowledge and cultural beliefs of parents. Further objective was to associate parental knowledge to proactive oral health behaviours.

## Materials and methods

### Study design

It was a comparative cross-sectional study and involved the completion of a questionnaire regarding parental knowledge and behaviours towards the oral health of their children. The research protocol was approved by the Ethics Committee of the School of Dentistry, National and Kapodistrian University of Athens (NKUA) (N447 approved on 06/11/2020), and the study was carried out in accordance with the Code of Ethics of the World Medical Association (Declaration of Helsinki). All parents who agreed to participate signed an informed consent.

### Sample selection

The sample consisted of parents seeking dental care for their children either at the Reception and Solidarity Center of the Municipality of Athens or the Postgraduate Paediatric Dentistry Department (NKUA). It was a convenience sample and included all parents who attended the aforementioned centres during the period between June 2021 and May 2022 and agreed to participate.

Inclusion criteria involved parents of children, aged 6 to 12 years, with a compromised medical history or mental impairment that might confound oral health behaviours, that could understand and speak Greek. The age range of 6–12 years was selected as it represents a key developmental period in which children begin to form independent health-related behaviours and attitudes whilst still being strongly influenced by parents.

### Data collection

Data were collected through a structured questionnaire that was distributed in a printed copy to all parents that agreed to participate. It consisted of 39 questions divided into five sections. First sections included questions regarding parental demographic characteristics (e.g. age, area of residence, educational level, occupation, and family income) and children’s medical and dental history. The section on parental behaviours involved questions regarding children’s everyday oral health habits (e.g. oral hygiene and dietary habits) and frequency and reasoning for dental visits. Knowledge was assessed with questions on the effect of oral health on general health and the aetiopathogenesis of common dental diseases through close-ended questions, in which one or more reasons for caries, dental plaque accumulation, and gingivitis formation could be chosen.

The questionnaire was a modified version of a previously used in Greek population (Agouropoulos 2012) and was piloted in a random sample of 20 parents (not included in the final sample) to ensure validity and reliability before application. It was completed, in the waiting room during the children’s scheduled appointments, only by one parent and for one child per family. Completion was anonymous and no specific information regarding participants’ personal details could be withdrawn.

### Statistical analysis

Patients’ demographics were presented using frequency tables. Data analysis regarding parental behaviours was based on SES, which was determined by family income, using the cut-off point of ≤ 1400 euros per month since it corresponds to the minimum country wage (No. 38866/21.4.2022 of the Ministry of Labor, as of 1 May 2022). Families with a monthly income of < 1400€ were considered as being of a low SES and those with incomes of between 1400 and 2500€ as medium. The upper limit of 2500 euros was chosen to keep the “medium” category from becoming too wide. If the threshold was to be extended to 3000 euros, the middle-income class might overlap with what many classifications regard as upper-middle class.

Parental knowledge was evaluated by comparing the frequency of positive answers regarding the association of oral and general health and the aetiopathogenesis of common dental diseases between groups. In an attempt to score in an arbitrary manner and categorise parental knowledge, each correct answer received one point, with a total possible score of five. A score of 0 to 1 indicated poor knowledge, whilst a score of 4 to 5 signified high parental knowledge.

Multivariate regression analysis was used to detect possible associations between low level of knowledge and specific parental characteristics and oral health behaviours. In the analysis, age variable was presented as binary using the mean calculated age of each parent included in the sample separately. The data collected were analysed using the Statistical Package for Social Sciences (SPSS Statistics for Windows, Version 27.0), IBM Corp. (2020, Chicago, IL, USA) and statistical significance was set at *p* < 0.05.

## Results

### Sample characteristics

Two-hundred-and-sixteen parents from 230 that were initially recruited, agreed to participate and successfully completed the questionnaire (response rate = 94%). From the participants, 111 belonged to the low socioeconomic group and had a mean chronological age of 42.6 years, whilst the remaining 105, with a mean chronological age of 43 years, were of medium SES.

Mothers from low SE groups in their majority had lived abroad until the age of 15 years (*n* = 77, 70%), had completed secondary education (*n* = 66, 59%), undertook home duties, and were not otherwise employed (*n* = 84, 76%). On the other hand, the majority of mothers from medium SE groups had lived in Greece until the age of 15 years (*n* = 93, 89%), had tertiary education (*n* = 62, 62, 59%) and were working in the public sector (*n* = 45, 43%). All differences were considered significant (Table 1).

**Table 1:** Demographic characteristics of parents according to socioeconomic group

		Low SE Group (N = 111)	Medium SE Group (N = 105)	
		Mean (s.d.) N (%)	Mean (s.d.) N (%)	*p value*
*Age (years)* Mother		39.7 (5.9)	40.5 (3.9)	0.29*
Father		45.4 (7.7)	45.5 (4.9)	0.88*
*Area of residence* Mother	Greece Abroad	34 (30) 77 (70)	93 (89) 12 (11)	0.02**
Father	Greece Abroad	37 (34) 74 (66)	94 (90) 11 (10)	0.01**
*Highest educational level* Mother	Primary Secondary Tertiary	23 (21) 66 (59) 23 (21)	0 (0) 43 (41) 62 (59)	0.03**
Father	Primary Secondary Tertiary	20 (18) 76 (68) 15 (14)	5 (5) 48 (45) 52 (50)	0.02**
*Occupation* Mother	Private sector Public sector Unemployed/Housewives	1 (1) 26 (23) 84 (76)	34 (32) 45 (43) 26 (25)	0.03**
Father	Private sector Public sector Unemployed	83 (75) 10 (8) 19 (17)	79 (75) 26 (25) 0 (0)	0.54**

*χ^*2*^ and **Fisher’s exact test

Distribution of the above variables amongst fathers of the two groups was similar, with fathers from higher SE groups presenting significantly more affluent areas of residency and higher levels of education (*p* < *0.05*).

### Parental behaviours

Non-significant differences were calculated regarding supervision and frequency of brushing and use of fluoridated toothpaste (100% in both groups), with parents from both groups showing favourable behaviours towards daily oral hygiene habits (Table 2). Significant differences were reported for consumption of sugary snacks, with parents from low SE groups reporting daily consumption, as compared to a less frequent one (× 4–5/week) for parents from the medium group (*p* = *0.04*). Significant differences were also calculated when reasoning for dental visits was considered, with parents from low SE groups reporting visiting the dentist only in cases of pain as compared to those from the medium group that reported prevention as the main reason for scheduled dental visits (*p* = *0.01*).

**Table 2:** Distribution of parental behaviours towards daily oral habits according to their socio-economic background

	Low socio-economic group N (%)	Medium socio-economic group N (%)	*p-value*
*Frequency of brushing* Twice per day Once per day Rarely	42 (28) 70 (47) 36 (25)	28 (42) 39 (58) 0 (0)	0.06**
*Person brushing* Alone Supervised Parent	2 (1) 133 (90) 13 (9)	1 (2) 53 (79) 13 (20)	0.09**
*Meals per day* Up to 3 4–5 More than 6	17 (12) 106 (72) 25 (17)	9 (13) 53 (79) 5 (8)	0.18
*Frequency of sugary snacks* Up to × 3/week 4–5 times/week Everyday	17 (12) 15 (10) 116 (78)	17 (26) 6 (9) 44 (65)	0.04
*Frequency of dental visits* Twice/year Once/year Infrequently	53 (36) 48 (32) 47 (32)	39 (58) 24 (36) 4 (6)	0.01
*Reason for dental visits* Routine check-up Prevention Restorative treatment Pain	4 (4) 10 (9) 24 (21) 73 (66)	50 (48) 28 (27) 10 (10) 17 (17)	0.01**

*χ^*2*^ and **Fisher’s exact test

### Parental knowledge

Regarding parental knowledge, 64% (*n* = 71) of the parents from the low SE group and 89% (*n* = 94) of parents from the medium SE group reported that oral health can affect general health. Corresponding percentages for awareness of the effect of general health on oral health were 67% (*n* = 74) and 87% (*n* = 91) in the two groups, with both differences being marginally significant (*p* = *0.05*).

**Fig. 1:**
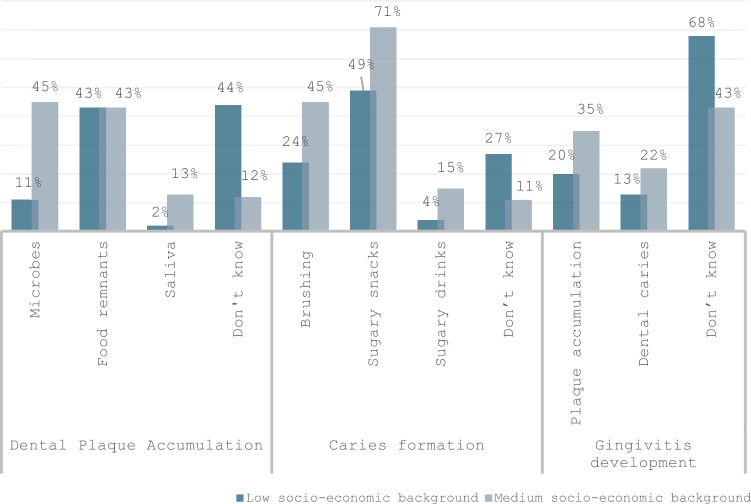
Distribution of answers regarding aetiopathogenesis of dental plaque accumulation and common dental diseases

Results on parental knowledge regarding aetiopathogenesis of dental plaque accumulation and common dental diseases showed great variability (Fig. 1). Parents from the low SE group reported in their majority that they do not know what causes plaque accumulation (*n* = 49/44%), and they reported sugary snacks (*n* = 54, 49%) as the main factor responsible for caries development. On the other hand, parents from the medium SE group showed increased knowledge, reporting microbes (*n* = 47, 45%) and food remnants (*n* = 45, 43%) for plaque accumulation and brushing (*n* = 47, 45%) and sugary snacks (*n* = 75, 71%) for caries development. Regarding gingivitis, parents in both groups, in their majority, were not able to correctly identify the main aetiological factors. Although it should be mentioned that > 1/3 of parents (*n* = 37, 35%) from medium SE backgrounds were able to identify plaque accumulation. The only significant differences were found for the presence of microbes for dental plaque accumulation (*p* = *0.03*) and brushing (*p* = *0.02*) and sugary snacks (*p* = *0.04*) for caries development.

**Fig. 2:**
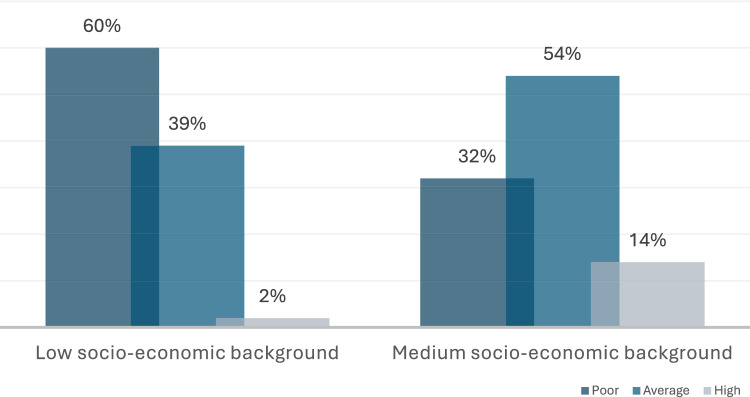
Distribution of level of parental knowledge regarding common dental diseases

Combination of the above parameters confirmed statistically significantly higher knowledge (*p* = *0.04*) regarding common dental diseases of parents from medium SE groups, with more than half of parents from low SE group (*n* = 67, 60%) having low level (Fig. 2).

### Association of knowledge with specific parental characteristics

Multivariate regression analysis for parental characteristics (Table 3) showed that the area where both parents lived up to the age of 15 years and the mother’s occupation were the factors significantly associated with decreased knowledge. Therefore, parents that have lived abroad and unemployed mothers have a greater chance of having a lower level of knowledge regarding oral health behaviours.

**Table 3:** Results of multivariate regression analysis for association of parental characteristics with low level of knowledge

	Unstandardised coefficients		Standardised coefficients			95% Confidence interval	
	B	SE	Beta	*t*	*p-value*	Lower Bound	Upper Bound
(Constant)	0.617	0.447		1.380	0.169	− 0.265	1.498
Age mother (years)	0.015	0.010	0.132	1.404	0.162	− 0.006	0.035
Age father (years)	0.007	0.009	0.082	0.860	0.391	− 0.010	0.024
Area Mother lived < 15 years (Greece/abroad)	− 0.361	0.186	− 0.294	− 1.947	0.053	− 0.728	0.005
Area Father lived < 15 years (Greece/abroad)	0.373	0.185	0.303	2.020	0.045	0.009	0.738
Educational level mother (primary/secondary/tertiary)	− 0.048	0.091	− 0.043	− 0.534	0.594	− 0.227	0.130
Educational level father (primary/secondary/tertiary)	0.015	0.075	0.017	0.206	0.837	− 0.132	0.163
Occupation mother (private/public/unemployed)	− 0.284	0.079	− 0.315	− 3.589	< 0.001	− 0.440	− 0.128
Occupation father (private/public/unemployed)	− 0.106	0.078	− 0.094	− 1.361	0.175	− 0.260	0.048
Income (euros)	− 0.013	0.124	− 0.010	− 0.104	0.917	− 0.258	0.232

Regarding association with parental behaviours, frequency of brushing and sugary snack consumption were significantly associated with decreased knowledge (Table 4). Parents with limited knowledge tend to report less pro-active behaviours such as infrequent toothbrushing and increased sugary snack consumption.

**Table 4:** Results of multivariate regression analysis for association of low level of knowledge to specific pro-active oral health behaviours

	Unstandardised coefficients		Standardised coefficients			95% Confidence interval	
	B	SE	Beta	*t*	*p value*	Lower Bound	Upper Bound
(Constant)	0.518	0.200		2.591	0.010	0.124	0.912
Person brushing (alone/supervised/parent)	− 0.011	0.066	− 0.012	− 0.170	0.865	− 0.141	0.118
Frequency brushing (twice/once/rarely)	− 0.121	0.044	− 0.199	− 2.734	0.007	− 0.208	− 0.034
Number of meals (3/4–5/ > 6)	− 0.047	0.080	− 0.040	− 0.594	0.553	− 0.204	0.110
Frequency of sugary snacks × 3/week/ × 4–5/week/everyday)	0.125	0.056	0.155	2.233	0.027	0.015	0.235
Frequency of dental visits (6-moths/12-moths/infrequently	− 0.056	0.053	− 0.078	− 1.057	0.292	− 0.160	0.048
Reasoning for dental visits (prevention/restoration/pain)	0.027	0.026	0.075	1.059	0.291	− 0.023	0.077

## Discussion

The present study aimed at assessing oral health knowledge and behaviours of parents from different socio-economic backgrounds and correlate knowledge with proactive behaviours to identify possible risk factors for poor dental behaviours. Despite the self-reported data that might introduce potential for recall and social desirability biases, it is one of the very few studies in the literature that has successfully highlighted significant disparities. Parents from medium SES demonstrate higher levels of awareness about the relationship between oral and general health, as well as better knowledge of the aetiopathogenesis of common dental diseases. A positive association was also shown for the area of residency up to the age of 15 years and maternal occupation with oral health knowledge, and a low level of knowledge with infrequent brushing and improper dietary habits such as frequent snacking.

Specifically, regarding oral health behaviours, parents from low socio-economic backgrounds, despite reporting adequate daily oral care, including frequent and supervised brushing, reported a higher frequency of sugary snack consumption, highlighting that dietary habits may further exacerbate disparities in oral health. Many studies have confirmed the increased snacking, including non-nutritious and sugary foods and drinks, of children from lower socio-economic backgrounds (Gangrade et al. 2021; Stephens et al. 2011; Craig et al. 2010). This could be explained by the fact that members of these families differ in their education and food culture, which is also combined with limited knowledge regarding hidden sugar contents of commonly consumed goods. Along the same line, healthy foods can often be more expensive, and families in poverty have entrenched living and food choices (Petrauskienė et al. 2015), making sugary snacks an easy choice.

Within the contents of lack of proper oral health behaviours, parents from low socio-economic backgrounds in the present study also reported infrequent dental visits and mainly for emergency treatment. This reactive approach to dental health, contributing to delayed treatment-seeking behaviour, is consistent with findings from other studies and is attributed to financial constraints as well as the lack of awareness concerning the benefits of preventive care (Singh et al. 2019). In many countries, like Greece, where the public health insurance system does not cover dental expenses, parents from disadvantaged backgrounds may struggle to prioritise oral health needs, as they are often preoccupied with other pressing family and caregiving responsibilities.

In the literature, reactive oral health behaviours have also been related to limited health literacy. This association suggests a lack of ability to comprehend and implement information related to oral health and hinders the adoption of self-care practices and timely access to dental services (Chakraborty et al. 2025). This is reflected in our study by the lower awareness regarding the impact of oral health on general well-being of parents from low socio-economic backgrounds. This knowledge gap is mainly attributed to the lack of effective health practitioner–patient communication, which is underlined by most patients reporting receiving inadequate information. This reflects the oral health disparities, as parents may not take the appropriate actions and the necessary precautions for their children if they are unaware of the risks involved (Mattila et al. 2005). It also reflects their limited access to healthcare services, which in some cases is related to the lifestyle of vulnerable groups and the possible language barrier that these families might face. It is worth noting that in the present study more than half of parents from low socio-economic backgrounds have lived abroad until the age of 15 years, a fact that can directly affect both knowledge and behaviours towards oral health. Although it should be mentioned that data regarding the area of residency could not be retrieved, limiting the interpretation of the results. The term “abroad” encompasses a wide range of contexts, and parents may have lived in either highly affluent, high-income countries or in less developed, low- or middle-income countries. These environments differ substantially in terms of healthcare access, cultural norms, dietary patterns, and oral health practices and may directly affect the way parental attitudes and practices have been shaped.

Limited oral health literacy may also reflect poor knowledge on the aetiopathogenesis of common dental diseases. Results from the present study showed that unlike parents from low socio-economic backgrounds, parents from medium socio-economic backgrounds were able to correctly name the presence of microbes as aetiological factors for dental plaque accumulation, and the frequency of brushing and sugary snacking for caries development. This can be attributed to the fact that they may engage more frequently with medical professionals, such as paediatricians and dentists, which is important for spreading knowledge about oral health literacy (Petersen and Kwan 2011). Intensive counselling and motivational interviews can increase understanding of the basis of oral diseases, which can be reflected in improved parental behaviours and oral hygiene practices. Although it should be noted that a gap persists between what parents know and what they finally practice.

In the development and establishment of positive behaviours towards oral health, mothers have been shown to play the most significant role. Results from the present study indicated that mothers that have lived abroad until the age of 15 years and unemployed mothers had significantly lower knowledge scores. Both factors can lead to a cycle of decreased opportunity and knowledge acquisition for the mother, which can, in turn, influence the development of proper behaviours towards oral health. Economic stress associated with unemployment and the psychological and social impact of factors associated with being foreign-born may influence oral health knowledge, though in an indirect way (Grandlund et al. 2022). This indirect relationship is in consistency with previous studies reporting the fundamental role of acculturation, the process of changing and integrating in time values and behaviours from another culture (Cruz et al. 2004).

On the other hand, results from the present study failed to indicate a positive correlation between maternal education levels and oral health knowledge. This is in agreement with some studies (Azimi et al. 2018) but in contrast with others reporting that mothers with a higher education level had significantly better oral health-related knowledge (Abanto et al. 2024; Bamashmous et al. 2024). Similarly, Diel et al. (2022) reported that higher maternal education was linked to a greater propensity to care for their children's primary dentition, even in the absence of pain. It is reasonable that mothers with a higher level of education can access and filter sources to find oral health information and integrate tools into broader educational frameworks to enhance awareness and knowledge about children's oral health and at the same time recognise the necessity of teaching their children (Kashani et al. 2024). The fact that in the present study most participants had at least a basic education may be one of the reasons that can justify the reported differences. Additionally, the questions were formulated to be basic and easily answered by the participants so that even less-educated parents could score well.

The findings of the present study confirmed the disparity between socioeconomic groups in terms of dental health poverty and provide practical knowledge for dental public health professionals as it highlighted population groups who would benefit from oral health preventative interventions. Particularly for disadvantaged families, implementing community-based programmes, improving access to affordable dental care, and integrating oral health education into broader public health campaigns could contribute to closing the gap in oral health discrepancies (Anyikwa & Ogwo, 2025). To provide parents from diverse socioeconomic and cultural backgrounds with pertinent and useful oral health information, it is imperative that healthcare providers get training in effective communication techniques.

### Strengths and limitations

Regarding the strengths of the study, it is worth mentioning the substantial sample sizes in both groups. Since there is a lack of adequate studies in the literature, the present study offered valuable information for the vulnerable population of parents from low socioeconomic backgrounds. The main limitation was that SE background was solely related to current family income, which may not accurately capture the family’s historic SES, indicating that the family’s present financial situation may not reflect the conditions in which the parents themselves were raised or the socioeconomic environment that shaped their early life experiences. Because past socioeconomic circumstances can influence long-term attitudes, behaviours, and health outcomes, relying solely on current income may misrepresent the broader SES context relevant to the study.

Another limitation is the use of a convenience sample drawn from individuals already seeking care at dental centres, which might imply selection from participants with more proactive health-seeking behaviours than the general population. Finally, being a questionnaire-based search, only Greek-speaking individuals were included, introducing possible selection and reporting bias, especially given the high rate of geographic mobility observed in the lower socio-economic group of parents.

## Conclusions

To conclude, findings from the present study reported that

- parents from a low socio-economic background showed significantly worse oral health behaviours, with more frequent sugary snacking, infrequent dental visits, and poor knowledge regarding common aetiological factors for dental plaque accumulation and caries development.
- poor knowledge regarding oral health was associated with the area where both parents lived up to the age of 15 years and mother’s occupation, and with the frequency of brushing and sugary snack consumption.

## Data Availability

The data that support the findings of this study are available from the corresponding author upon reasonable request.
